# Expression of *NORAD* correlates with breast cancer aggressiveness and protects breast cancer cells from chemotherapy

**DOI:** 10.1016/j.omtn.2023.08.019

**Published:** 2023-08-18

**Authors:** Catarina Alves-Vale, Ana Maria Capela, Carlota Tavares-Marcos, Beatriz Domingues-Silva, Bruno Pereira, Francisco Santos, Carla Pereira Gomes, Guadalupe Espadas, Rui Vitorino, Eduard Sabidó, Paula Borralho, Sandrina Nóbrega-Pereira, Bruno Bernardes de Jesus

**Affiliations:** 1 Instituto de Medicina Molecular João Lobo Antunes, Faculdade de Medicina, Universidade de Lisboa, Av. Professor Egas Moniz, 1649-028 Lisboa, Portugal; 2 Hospital CUF Descobertas, CUF Oncologia, 1998-018 Lisbon, Portugal; 3 Department of Medical Sciences and Institute of Biomedicine – iBiMED, University of Aveiro, 3810-193 Aveiro, Portugal; 4 i3S – Instituto de Investigação e Inovação em Saúde, Universidade do Porto, Porto, Portugal; 5 IPATIMUP – Instituto de Patologia e Imunologia Molecular da Universidade do Porto, Porto, Portugal; 6 Center for Genomic Regulation, Barcelona Institute of Science and Technology (BIST), Barcelona, Spain; 7 Universitat Pompeu Fabra, Barcelona, Spain; 8 Faculdade de Medicina, Universidade de Lisboa, Av. Professor Egas Moniz, 1649-028 Lisboa, Portugal

**Keywords:** MT: Non-coding RNAs, *NORAD*, DNA damage, breast cancer, chemotherapy, H2Ax, triple-negative breast cancer

## Abstract

The recently discovered human lncRNA *NORAD* is induced after DNA damage in a p53-dependent manner. It plays a critical role in the maintenance of genomic stability through interaction with Pumilio proteins, limiting the repression of their target mRNAs. Therefore, *NORAD* inactivation causes chromosomal instability and aneuploidy, which contributes to the accumulation of genetic abnormalities and tumorigenesis. *NORAD* has been detected in several types of cancer, including breast cancer, which is the most frequently diagnosed and the second-leading cause of cancer death in women. In the present study, we confirmed upregulated *NORAD* expression levels in a set of human epithelial breast cancer cell lines (MDA-MB-231, MDA-MB-436, and MDA-MB-468), which belong to the most aggressive subtypes (triple-negative breast cancer). These results are in line with previous data showing that high *NORAD* expression levels in basal-like tumors were associated with poor prognosis. Here, we demonstrate that *NORAD* downregulation sensitizes triple-negative breast cancer cells to chemotherapy, through a potential accumulation of genomic aberrations and an impaired capacity to signal DNA damage. These results show that *NORAD* may represent an unexploited neoadjuvant therapeutic target for chemotherapy-unresponsive breast cancer.

## Graphical abstract

**Figure:**
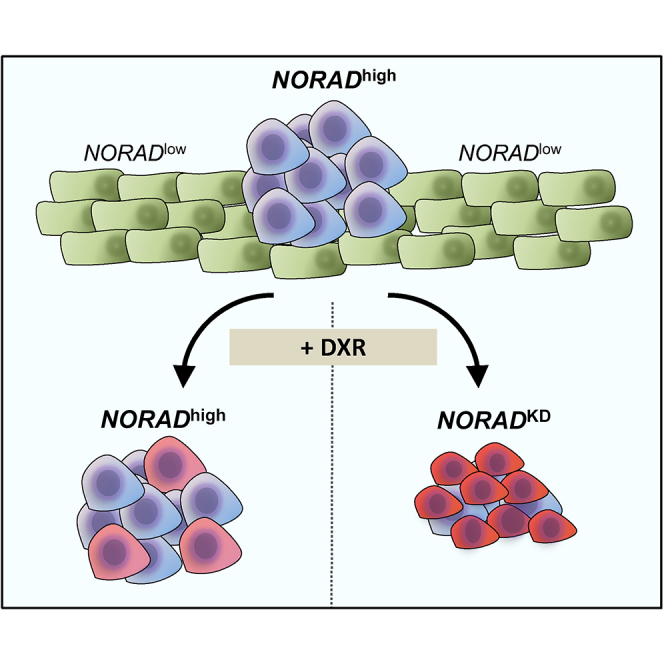

## Teaser

Bernardes de Jesus and colleagues uncover how *NORAD* downregulation sensitizes triple-negative breast cancer cells to chemotherapy, through an impaired DNA damage response, highlighting how *NORAD* may represent an unexploited therapeutic target for breast cancer.

## Introduction

The Human Genome Project provided scientists and society with transformational insights into the intriguing complexity of the transcriptome of human cells.^1^ Long non-coding RNAs (lncRNAs) constitute the broadest class of non-coding RNAs, displaying a tissue-specific spatiotemporal expression profile,^2^ with numerous biological roles identified, spanning from development to aging, in both normal and pathological conditions, such as age-related diseases.^3^^,^^4^^,^^5^ The study of differential gene expression in cancer has led to the identification of thousands of associated lncRNAs^2^ involved in several cancer hallmarks including genomic instability, tumor-promoting inflammation, and evasion of immune detection.^6^^,^^7^ LncRNA *NORAD*^8^ is a 5.3 kb transcript, annotated as *LINC00657*, localized on chromosome 20 (20q11.23).^9^ *NORAD* shows strong evolutionary conservation and is widely expressed in human tissues and cell lines.^9^^,^^10^ *NORAD* seems to play a crucial role in the maintenance of genomic stability: its inactivation triggers chromosomal instability in previously karyotypically stable cell lines, and expression levels of this lncRNA seem to increase after inducing DNA damage with doxorubicin.^9^ One of the possible mechanisms involves *NORAD* sequestering PUMILIO-1 and PUMILIO-2 RNA-binding proteins that target mRNAs and reduce their stability.^9^^,^^11^^,^^12^^,^^13^ PUMILIO interaction seems to be mediated by SAM68, an abundant and multifunctional cell-cycle-regulated RNA-binding protein.^14^ Therefore, *NORAD* levels directly influence the availability of PUMILIO to downregulate a set of factors involved in mitosis, DNA repair, and DNA replication.^9^ Nonetheless, many genes regulated by *NORAD* are not PUMILIO targets, suggesting that other mechanistic events are involved, such as miRNA sponging. Considering the complexity of the *NORAD* network, *NORAD* appears to have a dual effect depending on the tumor type.^9^^,^^15^^,^^16^ Among those interactors, there was shown to be enrichment of DNA damage response (DDR)-associated proteins, mitotic cell cycle and minichromosome maintenance (MCM) complex.^17^ Some previously identified *NORAD* interactors are nucleosome assembly protein 1-like 4 (NAP1L4),^17^ a histone chaperone^18^ involved in the chromatin assembly step related to DNA replication and repair.^19^ The nucleosome assembly protein 1 is an H2A-H2B chaperone,^20^ preventing excessive accumulation of these chromatin marks.^21^ *NORAD* also binds to the RNA binding motif protein X-linked (RBMX), which participates in the DDR, inducing the assembly of the *NORAD*-activated ribonucleoprotein complex 1 nucleic complex, through RBMX, promoting genomic and chromosomal stability.^22^

The intrinsic resistance of neoplastic disorders to chemotherapy and targeted therapy represents a major clinical concern.^23^ The underlying causes of resistance can be attributed to intratumor heterogeneity,^23^^,^^24^ in part due to genomic instability.^25^ Chromosomal instability, a hallmark of cancer, is often associated with cancer progression, correlating with poor breast cancer prognosis.^26^ Paradoxically, by affecting cancer cell fitness, chromosomal instability may be exploited and have beneficial roles against cancer, namely in estrogen receptor (ER)-negative tumors, which were found to be associated with a better long-term survival when extreme levels of chromosome instability were present.^27^

Considering the correlation between *NORAD* and genome instability, as well as the contradictory effect of chromosomal instability in tumor progression, we investigate whether targeting *NORAD* could act synergistically with cytotoxic agents.^27^^,^^28^ Here, we demonstrate that downregulation of *NORAD* sensitizes human breast cancer cells to doxorubicin. *NORAD* expression was shown to be needed to signal the DNA damage after doxorubicin treatment. Our results underline the potential contribution of *NORAD* in chemotherapy-resistant cancer cells.

## Results

### *NORAD* is highly expressed in triple-negative breast cancer

Breast cancer is a heterogeneous disease and four main clinicopathological groups (luminal A-like, luminal B-like, HER2-positive (non-luminal), and triple-negative) are defined based on the expression of ERs, progesterone receptors (PRs), human epidermal growth factor receptor 2 (ERBB2/HER2), and Ki67.^29^

**Figure 1:**
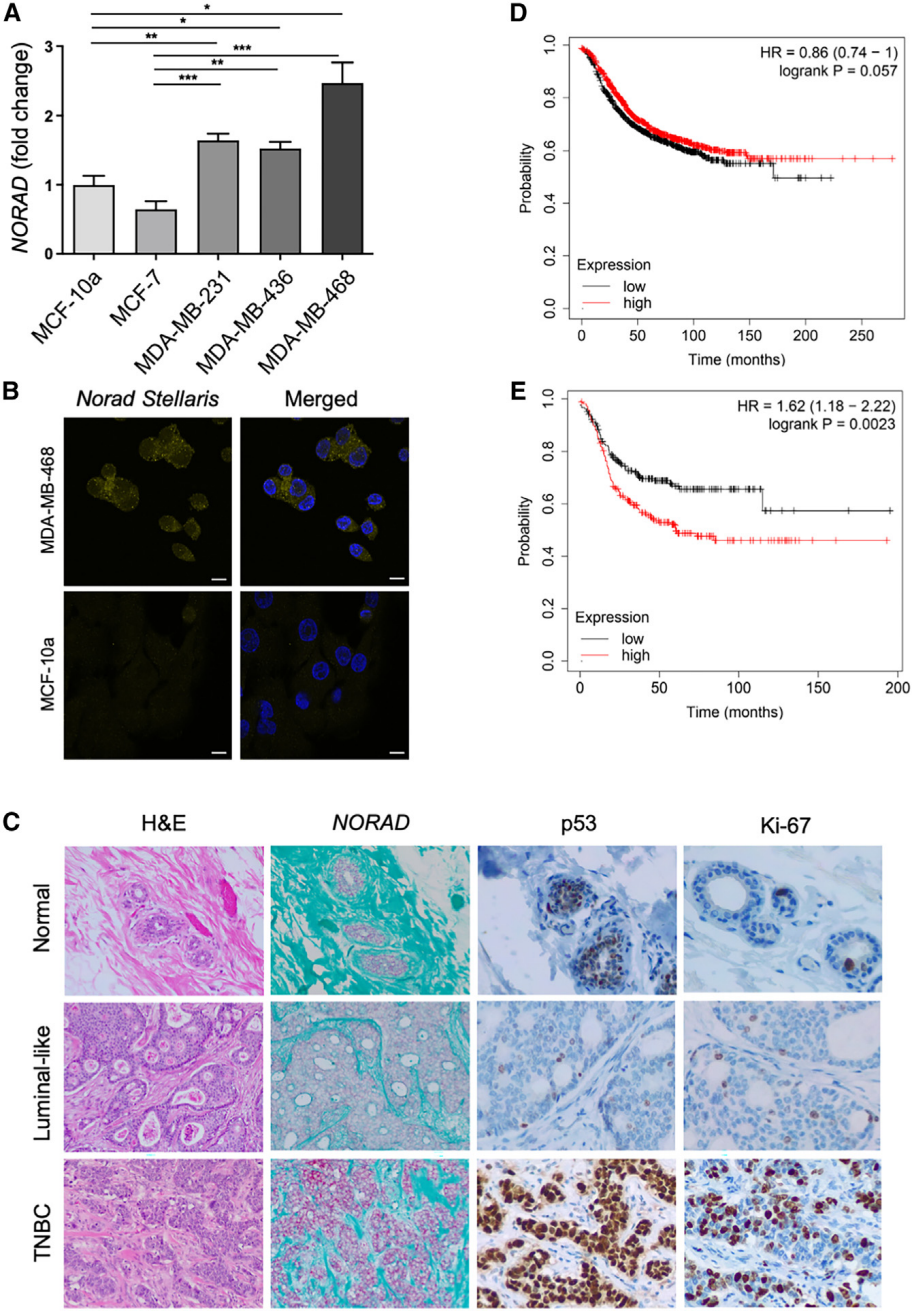
*NORAD* characterization (A) *NORAD* mRNA basal levels in human epithelial breast cancer cell lines (qRT-PCR) (n = 3). (B) *NORAD* subcellular localization in the MDA-MB-468 and MCF10a cell lines (smRNA FISH). Scale bar, 10 μm). (C) Higher *NORAD* expression (by RNA *in situ* hybridization, RNAscope) in triple-negative breast invasive carcinoma (TNBC) (lower) compared with luminal-like invasive (cribriform) carcinoma (middle) and human normal mammary tissue (upper) (n = 1, each); strong and diffuse p53 immunostaining and higher proliferative index (Ki67) in TNBC; hematoxylin and eosin (H&E) staining and *NORADin situ* hybridization at 100×; p53 and Ki67 immunostaining at 200× magnification. (D and E) Correlation between *NORAD* expression and prognosis of breast cancer patients (Kaplan-Meier Plotter): relapse-free survival irrespective of breast cancer subtype (A) and for basal-like breast cancer (E) (curves show the probability of survival over time and are colored based on the *NORAD* levels, the x axis represents time in months, and the y axis represents the proportion of patients who are still alive without relapse). For statistical analysis we used one-way ANOVA with a control condition for multiple comparisons. No symbol, p > 0.05, ∗p < 0.05, ∗∗p < 0.01, ∗∗∗p < 0.001.

Initially, we determined the basal mRNA levels of *NORAD* in a set of human breast cancer cell lines (MCF-7, MDA-MB-231, -436, and -468) and in a non-malignant human mammary epithelial cell line (MCF-10A) by real-time quantitative reverse transcriptase polymerase chain reaction (qRT-PCR). We observed that MDA-MB-231, -436, and -468 cell lines, corresponding to triple-negative breast cancer (TNBC), express higher levels of *NORAD*. On the other hand, the luminal A-like subtype MCF-7 cell line expresses *NORAD* at comparable levels with control MCF-10A cell line (Figure 1A). The same pattern could be detected when we compared *NORAD* levels through fluorescence *in situ* hybridization (FISH) using Stellaris-specific probes (Figure 1B).

TNBC, considered the most aggressive breast cancer subtype, is defined by the absence of ER, PR, and HER2, detected through immunohistochemical staining, which limits targeted hormonal therapeutic options.^30^ On the contrary, luminal-like breast cancer is characterized by the expression of hormonal receptors (ER and/or PR) and a more indolent clinical behavior. Therefore, our findings suggest that high expression of *NORAD* may be indicative of a more aggressive form of breast cancer. These results align with those obtained by analyzing formalin-fixed paraffin-embedded (FFPE) human breast samples using single-molecule RNA *in situ* hybridization (RNAscope), which revealed higher *NORAD* expression in TNBC compared with the luminal-like tumor and normal mammary epithelium (Figure 1C). Neither patient had received previous chemotherapy. Despite the low level of evidence to recommend p53 immunohistochemical assessment for routine use, some studies suggest that abnormal staining correlates with aggressiveness features.^31^ The p53 expression pattern strongly differed between the two neoplasms, being heterogeneous (“wild-type” pattern) in the luminal-like tumor, with strong and diffuse staining (overexpression/accumulation, “mutated-type” pattern) in TNBC. This observation is concordant with the higher frequency of *TP53* mutations in tumors classified as basal-like, which are a subtype of TNBC defined by specific gene expression patterns (Figure 1C).

Next, we asked how *NORAD* expression levels correlated with cancer patients’ outcome. We used the Kaplan-Meier Plotter Tool to correlate *NORAD* levels with prognosis of breast cancer patients. The KMPlotter database incorporates several gene expression profiles; breast cancer samples are stratified into high- and low-expression groups using the median gene expression level as a cutoff.^32^ Considering all breast cancer subtypes as a group, *NORAD* levels do not correlate with relapse-free survival (n = 2032, p = 0.057) (Figure 1D) nor overall survival (n = 943, p = 0.25) (Figures S1A and S1B). However, there is a statistically significant negative correlation between *NORAD* levels and relapse-free survival (n = 953, p = 0.002) when considering, in isolation, basal-like tumors (defined by PAM50 genetic profiling) (Figure 1E). Even though high *NORAD* levels are correlated with a lower relapse-free survival in poorly differentiated (grade 3) tumors (n = 417, p = 0.026), no association between *NORAD* expression and survival for the remaining tumor subtypes (luminal or HER2+) was found (Figures S1C–S1F). Therefore, high *NORAD* levels seem to be a survival prognostic factor specifically for patients with TNBC.

### *NORAD* knockdown affects relevant tumor-specific phenotypes and sensitizes TNBC cells to chemotherapy

**Figure 2:**
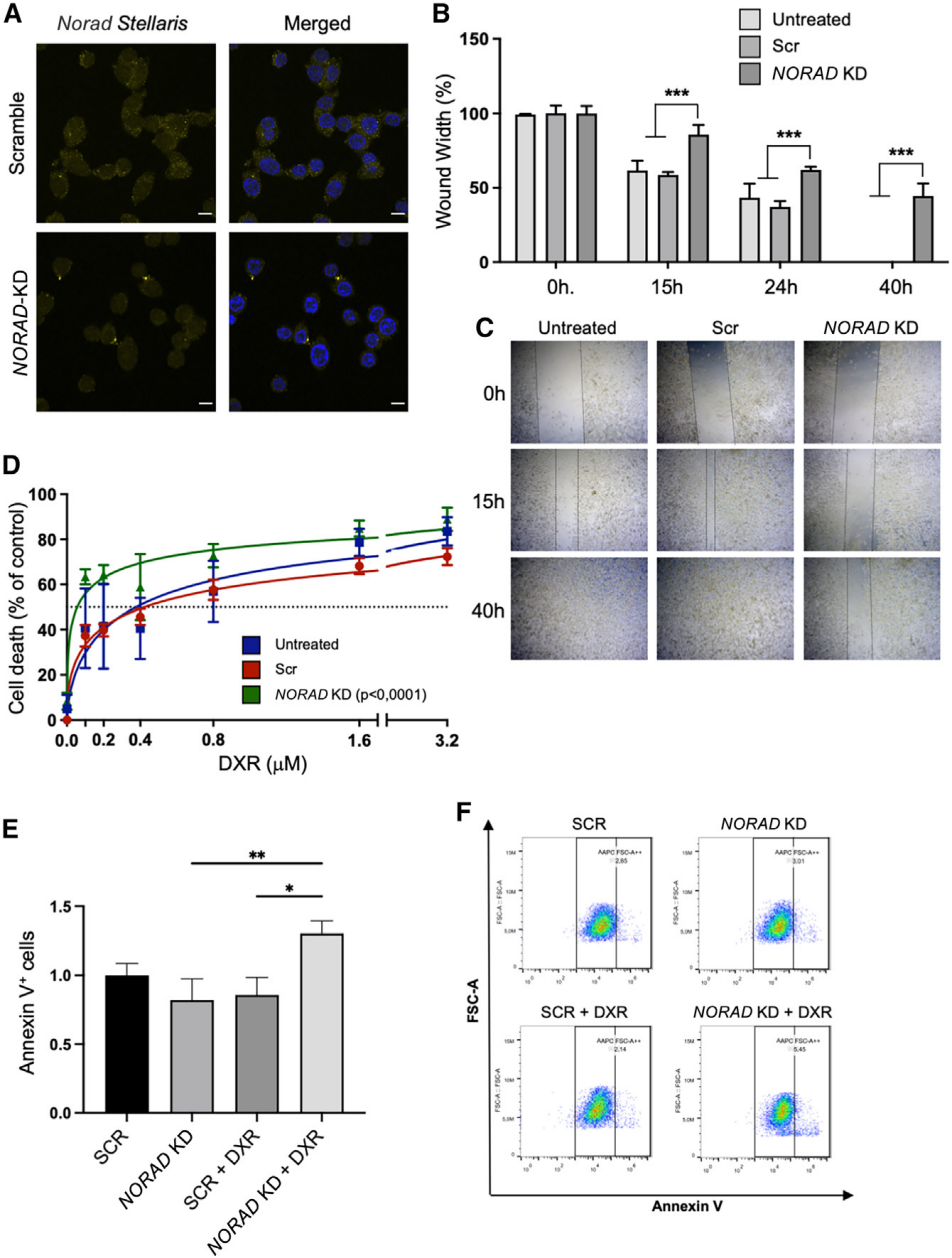
*NORAD* KD effects on tumor-relevant phenotypes (A) *NORAD* levels in the MDA-MB-468 cell line (smRNA FISH) treated with control siRNA + LNA or *NORAD*-specific siRNA + LNA. (B and C) *NORAD* KD effect on cell migration in the MDA-MB-231 cell line (wound healing assay); (B) is the gap quantification at the indicated time points (n = 3), (C) is a representative image of the wound healing. (D) *NORAD* KD sensitizes cells to doxorubicin (DXR), measured through the alamarBlue reduction assay (n = 3). (E and F) *NORAD* KD and doxorubicin effects on cell apoptosis in the MDA-MB-231 cell line (n = 3) as measured through the increase in Annexin V^+^ cells and as visualized in the representative plots (F). No symbol, p > 0.05, ∗p < 0.05, ∗∗p < 0.01, ∗∗∗p < 0.001.

To unveil the role of *NORAD* in breast cancer, we used LNA GapmeRs and siRNAs targeting both the nuclear and cytoplasmic fractions of *NORAD*. Two LNA GapmeRs that target different regions of *NORAD* were tested individually and in combination in the MDA-MB-231 and MDA-MB-468 (Figures S2A–S2C) at final concentrations of 25 and 50 nM. We observed the most significant and consistent reduction of *NORAD*, confirmed by smRNA FISH in MDA-MB-468, using LNA GapmeRs in combination with siRNAs, at a final concentration of 25 nM, with an interval of 24 h between transfections (Figure 2A).

Characterization of breast cancer cells was performed 48 h after *NORAD* downregulation, the time at which we detected lower *NORAD* levels. In addition, it has been demonstrated previously that 24 h after *NORAD* knockdown (KD) is not sufficient to affect PUMILIO-targeted mRNAs related to cell cycle and mitosis.^12^ We first tested whether *NORAD* affected the capacity of TNBC cells to migrate through the wound healing assay. After *NORAD* KD, a reduction in the migration rates of cells was evident, in comparison with the controls (Figures 2B, 2C, and S3). This result supports the role of *NORAD* in tumorigenesis, since invasiveness is one of the hallmarks of cancer.^6^^,^^7^ Considering the higher expression levels of *NORAD* in aggressive tumors, we addressed whether this transcript might be associated with chemoresistance. We tested the chemotherapeutic agent doxorubicin, an anthracycline commonly used in breast cancer treatment,^33^^,^^34^ which disrupts topoisomerase II-dependent DNA repair and mitochondrial function.^35^ Initially, we determined the half-maximal inhibitory concentration (IC_50_) for MCF-10A (Figure S4A) and MDA-MB-231 (Figure S4B) cell lines, confirming the higher values for non-malignant human mammary epithelial cells and demonstrating the chemotherapy selectivity toward cancer cells with higher proliferation rates. Then, we evaluated the effects of combining *NORAD* KD with chemotherapy. We observed a reduction in doxorubicin IC_50_ upon *NORAD* KD through alamarBlue cellular viability assay (Figures 2D and S5, IC_50_ shifted from 0.3779 to 0.05680 μM), indicating that *NORAD* KD sensitizes breast cancer cells to chemotherapy. This was accompanied by an increase in Annexin V^+^ cells (Figures 2E and 2F), something previously observed after doxorubicin treatment.^36^

### *NORAD* dowregulation impairs DNA damage pathways

**Figure 3:**
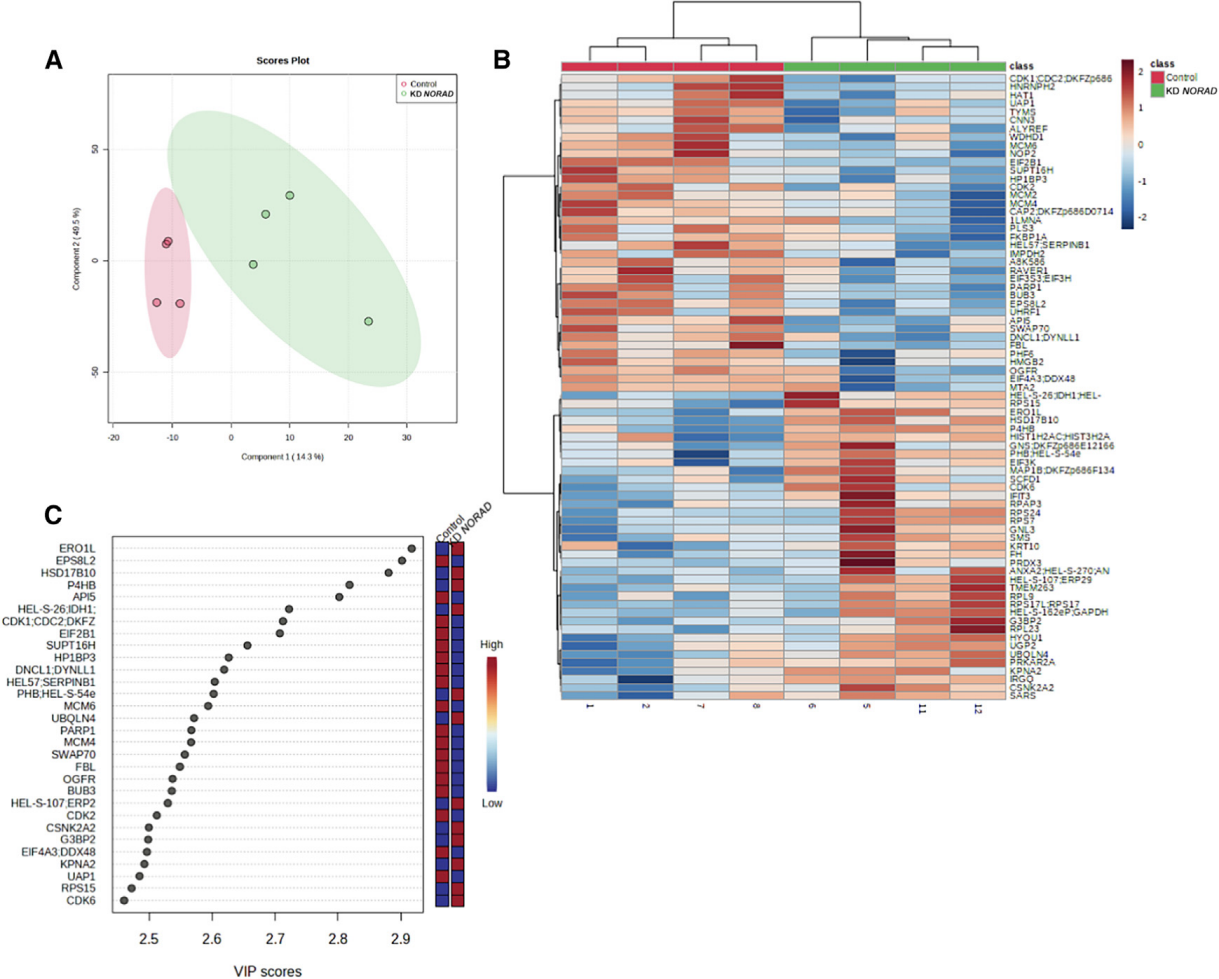
*NORAD* KD alters the proteome balance toward genetic instability (A) Partial least-squares discriminant analysis (PLS-DA) of the different variables tested (control vs. *NORAD* KD). (B) Heatmap showing the hierarchical clustering of the top 100 hits contributing to the separation of the variables control vs. *NORAD* KD. (C) Variable importance in projection (VIP) scores of the top 30 genes between the conditions control vs. *NORAD* KD.

To identify *NORAD*-associated proteins that could be mediating the increased sensitivity to doxorubicin we performed liquid chromatography-tandem mass spectrometry (LC-MS/MS) for quantitative comparison between groups (*NORAD* wild-type vs. *NORAD* KD) in the MDA-MB-231 cell line. Four independent conditions were individually analyzed. Whole proteome analysis by LC-MS/MS retrieved 4,167 unique proteins with at least two unique peptides. Of all proteins detected, 1,464 were common to all the conditions studied, leading to 35% of common proteins. Partial least-squares discriminant analysis (PLS-DA) was used to visualize group separation based on proteome datasets by means of dimensionality reduction. PLS-DA showed a clear separation of the experimental groups (Figure 3A). *NORAD* KD appeared, however, to increase the heterogeneity of the proteome, which was probably related with the KD efficiency (Figure 3A). To find quantitative patterns between the experimental groups, comparative and grouped analysis was performed (Figure 3B). When looking at experimental groups, two main clusters were apparent with a different profile of proteins being either overexpressed or repressed in the different experimental conditions (adjusted p < 0.05). *NORAD* KD downregulated proteins that were predominantly involved in biological processes related with G1/S transition of mitotic cell cycle and DDR (Figure 3C). The analysis revealed a preponderant altered modulation of proteins involved in the regulation of DNA repair, chromatin remodeling, and epigenetic regulation (Figure S6), suggesting that *NORAD* KD could affect the sensitivity of the MDA-MB-231 cells to doxorubicin by modulating the activity of proteins involved in DNA repair and epigenetic regulation. One example is MCM protein 6 (MCM6), the levels of which strongly decrease after *NORAD* KD. MCM6 is involved in the initiation of DNA replication and is a strong predictor of survival in cancer patients,^37^ or ALYREF, a known interactor of *NORAD*,^22^ a factor associated with poor survival in breast cancer patients.^38^ The lower expression of some detected proteins could be moderately confirmed by qPCR, demonstrating that *NORAD* may regulate the level of these proteins by other pathways, not only at the transcriptional level (Figures S6B–S6D).

### *NORAD* in the response to DNA damage

**Figure 4:**
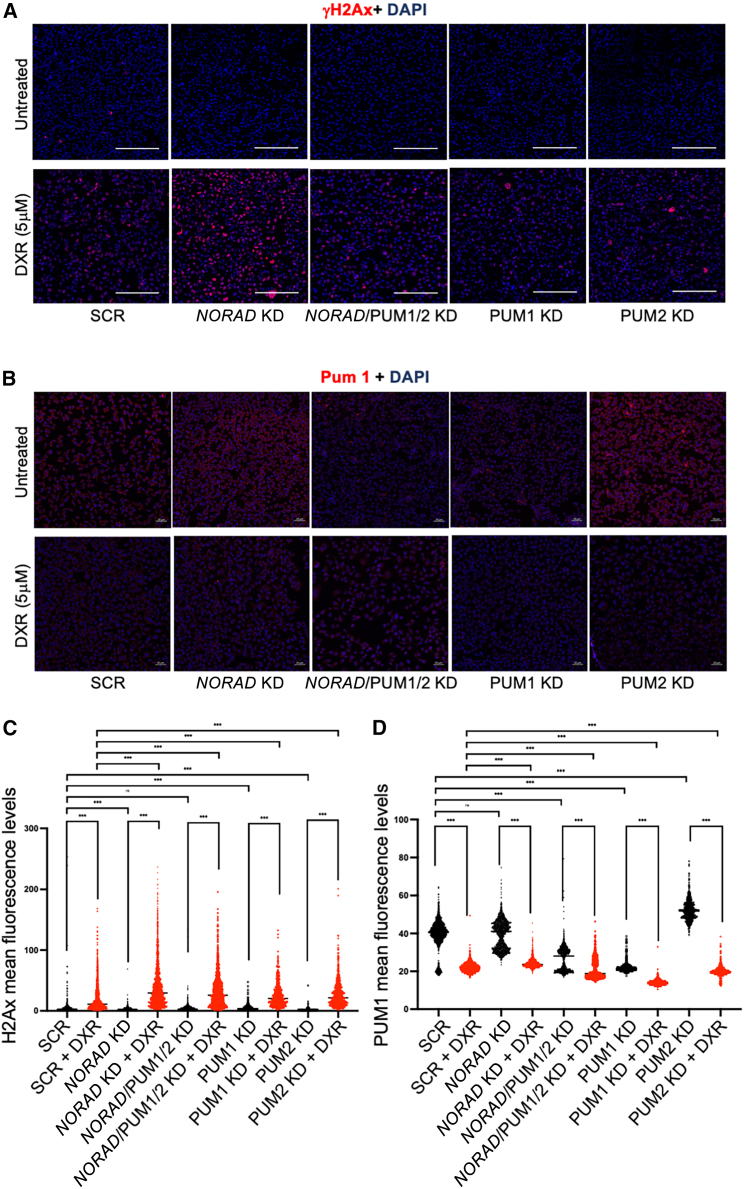
*NORAD* KD alters γH2Ax accumulation after DNA damage (A and B) Immunofluorescence (A) for γH2Ax and (B) for Pum1 in the depicted experimental conditions in the MDA-MB-231 cell line. Scale bars, 200 μm (A) and 50 μm (B). (C and D) Quantification of the signal corresponding to γH2Ax (C) and Pum1 (D) in the depicted experimental conditions in the MDA-MB-231 cell line (see materials and methods). No symbol, p > 0.05, ∗p < 0.05, ∗∗p < 0.01, ∗∗∗p < 0.001.

**Figure 5:**
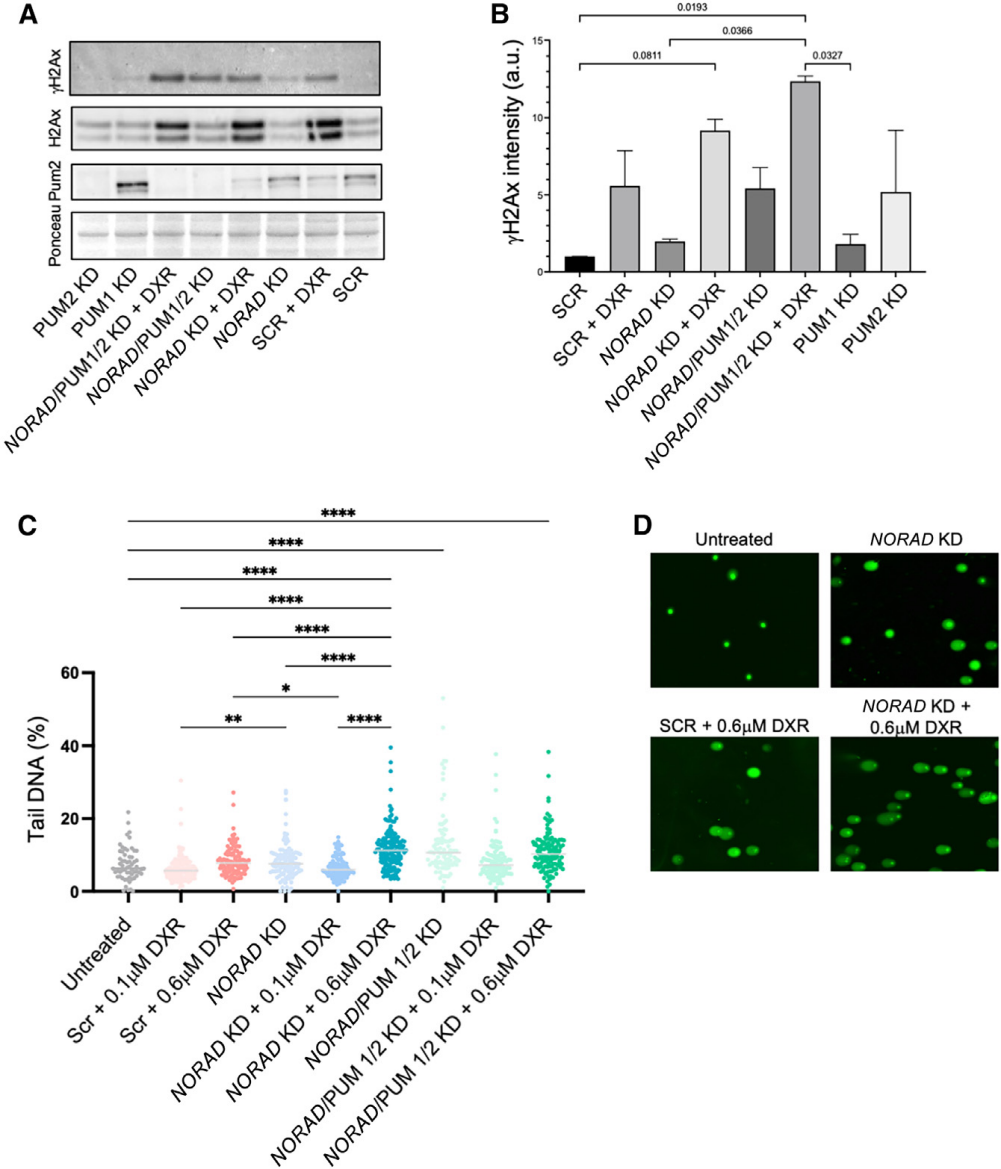
*NORAD* KD alters the DDR (A) Western blot analysis of γH2Ax, H2Ax, and Pum2 in the depicted experimental conditions in the MDA-MB-231 cell line. Ponceau was used as loading control. (B) Quantification of the western blot bands for the depicted proteins and conditions using the Ponceau band as loading control (n = 3). (C and D) DNA damage detection in the MDA-MB-231 cell line through comet assay (see materials and methods) in the depicted conditions. Quantification (C) and representative images (D) of the Comet assay is depicted. No symbol, p > 0.05, ∗p < 0.05, ∗∗p < 0.01, ∗∗∗p < 0.001.

In line with previous observations on the role of *NORAD* in DDR, and our own results supporting the sensitivity of *NORAD* KD cells to doxorubicin, we wondered how MDA-MB-231 or MDA-MB-468 cells with silenced *NORAD* would recognize and repair DNA lesions. Immediately after DNA double-strand breaks (DSBs) occur, histone H2AX is phosphorylated (γH2AX) mainly by ATM at C-terminal Ser136 and Ser139 residues,^39^ leading to signal amplification that ends with chromatin remodeling and recruitment of DNA repair proteins, such as BRCA1 and 53BP1. Using immunofluorescence (IF) we observed that *NORAD* KD resulted in an exacerbated accumulation of γH2AX in the MDA-MB-231 cells exposed to different concentrations of doxorubicin (Figures 4A, 4C, S7, and S8). This effect was not exacerbated when the Pumilio 1 and 2 proteins were concomitantly targeted with *NORAD*. Targeting Pumilio proteins separately (Figure S9) also resulted in some accumulation of γH2AX, although not equivalent to *NORAD* KD (Figures 4A and 4C), demonstrating a multifaceted role for Pumilio family of proteins in the setting of doxorubicin-induced DNA damage in breast cancer cells. Pumilio proteins have crucial roles in several cellular pathways, spanning mitosis and DNA repair. Whether DNA damage may induce Pumilio expression was also assessed by IF (Figures 4B and 4D). The presence of doxorubicin was shown to significantly decrease the presence of Pumilio 1 proteins independently of *NORAD* in cancer cells. Interestingly, in the absence of Pumilio 2 there is a compensatory expression of Pumilio 1, previously observed in the context of stemness and embryogenesis.^40^ Still, this increased expression does not impact on the signaling of DNA damage (Figures 4A and 4C). To further explore these results we evaluated γH2AX, H2AX, and Pum1 expression by western blot (WB) (Figures 5A and 5B). Similarly to IF, we could detect an increased expression of γH2AX in the conditions where *NORAD* was absent. Interestingly, the same compensatory role of Pumilio could be observed, since Pum2 levels greatly increased when Pum1 was targeted (Figures 5A and 5B). Of note, *NORAD*/PUM1/2 KD has a comparable level of yH2AX levels as SCR + DXR alone by WB, showing an impact of these proteins on DDR.

To understand whether this increase in DNA damage signaling correlated with an accumulation of DNA breaks, Comet assay was performed to explore different cellular conditions. DNA damage is measured by the presence of DNA in the comet tail, indicative of DNA break intensity.^41^^,^^42^ As expected, DNA breaks have been detected in the presence of doxorubicin. KD of *NORAD* increased the amount of DNA damage at 0.6 μM of DXR (Figures 5C and 5D), supporting previous results showing an increased accumulation of γH2Ax or cell death in the *NORAD* KD condition. Again, the condition *NORAD*/PUM1/2 KD had an increased amount of DNA damage even in the absence of DXR.

**Figure 6:**
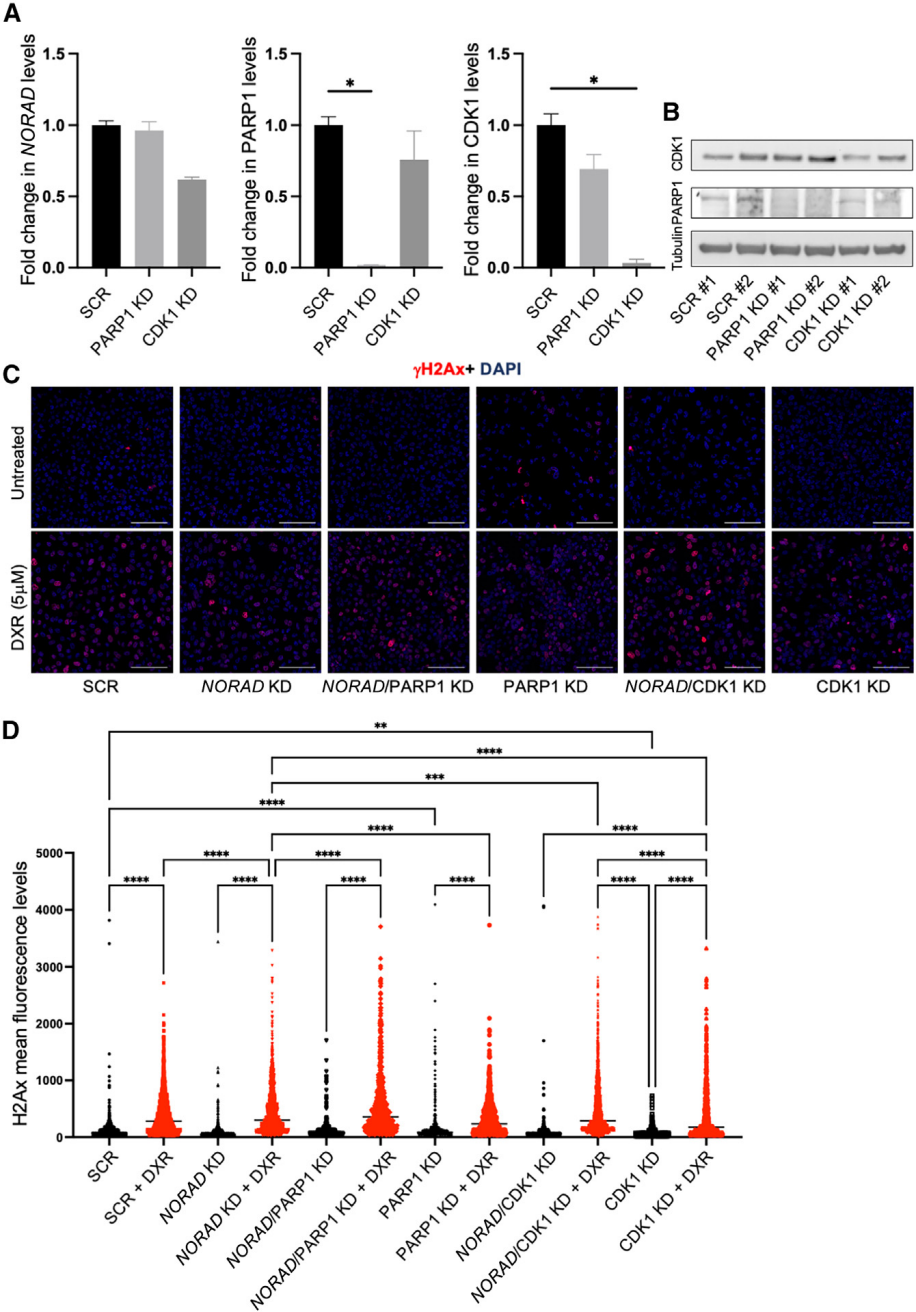
*NORAD* KD synergizes with PARP1 in the DDR (A) *NORAD*, PARP1, and CDK1 mRNA levels after KD of the conditions represented, using siRNAs (n = 3). (B) Western blot analysis of PARP1 and CDK1 in the depicted experimental conditions in the MDA-MB-231 cell line. Tubulin was used as loading control. (C) Immunofluorescence for γH2Ax in the depicted experimental conditions in the MDA-MB-231 cell line. Scale bar, 100 μm. (D) Quantification of the signal corresponding to γH2Ax in the experimental conditions represented in the MDA-MB-231 cell line (see materials and methods). No symbol, p > 0.05, ∗p < 0.05, ∗∗p < 0.01, ∗∗∗p < 0.001.

To further explore the involvement of the DDR proteins in the *NORAD*-mediated accumulation of γH2Ax we used siRNAs to KD (Figures 6A and 6B) two of the hits identified in the *NORAD*-associated proteome (LC-MS/MS experiment, Figure 3), namely PARP1 and CDK1, both proteins being extensively linked to cancer and DXR response.^43^^,^^44^^,^^45^^,^^46^^,^^47^ Although *NORAD* KD already impacted on the levels of PARP1 and CDK1 (Figure 3), siRNA-mediated KD showed a more consistent and severe reduction of their levels. Using IF we observed that *NORAD*/PARP1 KD, in the presence of DXR, resulted in a higher level of accumulation of γH2Ax, demonstrating the synergistic role of these two factors (Figures 6C and 6D). PARP1 alone, in the absence of DXR, increased the mean γH2Ax intensity (SCR, 105, to PARP1 KD, 189). Although CDK1-KD increased the 75th percentile of mean γH2Ax intensity (*NORAD* KD + DXR, 614; *NORAD*/PARP1 KD + DXR, 692; and *NORAD*/CDK1 KD + DXR, 692), the average intensity was not altered, probably due to an incomplete reduction of CDK1 levels, as depicted by WB (Figure 6B).

## Discussion

In this study, we reported that *NORAD* is overexpressed in breast cancer, conferring resistance to chemotherapy, and interfering with γH2AX signaling upon DNA damage.

It was described previously in esophageal, breast, lung, pancreatic, bladder, and colorectal cancers that *NORAD* functions as a potential oncogenic factor, suggesting that it may constitute a tumor biomarker, defining patient prognosis, predicting therapy response, and/or be used as a therapeutic target.^16^^,^^28^^,^^48^^,^^49^^,^^50^ Our results support this scenario where higher *NORAD* levels are associated with an aggressive breast cancer subtype (TNBC) and poor relapse-free survival of patients, while *NORAD* KD inhibits cancer cell viability and migration. Despite this association, it was also described previously in liver cancer that *NORAD* functions as a potential tumor suppressor.^51^ These opposing results may be explained by the distinctive interacting partners of *NORAD*, as it is known to sponge a myriad of miRNAs, albeit binding preferentially to PUMILIO proteins known to repress mRNAs involved in mitosis, DNA repair, and replication (e.g., PRC1, PARP1, and WDHD1),^9^^,^^12^ but also repress mRNAs involved in various cancer pathways (e.g., E2F3).^52^ Alternatively, SAM68 binds to conserved secondary structures immediately downstream of the PUMILIO response elements in *NORAD*.^12^ Of note, SAM68 is upregulated in several cancer types including breast cancer and could be involved in the deregulation of the AKT pathway.^53^ Interestingly, SAM68 also presents tumor suppressor-like activities as a transcriptional coactivator of p53.^14^ Nevertheless, it is still debated whether *NORAD* action is solely mediated through PUMILIO proteins. One example is the role of RBMX, a component of the DDR that may be mediating *NORAD* function in genomic (in)stability, whose role has been discussed by different authors.^13^^,^^22^ Given the complexity of *NORAD* and the different molecules that can associate with it, one would expect that different binding molecules may cooperate to different responses.^10^^,^^54^ Here, we demonstrate that the absence of PUMILIO did not synergize with *NORAD* in the intensity of γH2Ax signal or the levels of DNA damage after DXR. *NORAD* KD is somehow destabilizing the DDR complex, as supported by the LC-MS/MS data where we see, for instance, lower levels of PARP1, MCM6, or ALYREF (previously shown to be in a complex with *NORAD*^22^ and involved in carcinogenesis^38^). Whether PUMILIO may have a compensatory role in the response to DXR, in the *NORAD* KD scenario, later in time, or whether DXR may induce changes in the transcriptional program evading some of the PUMILIO regulated genes, is still unknown. It is known, however, that PUMILIO proteins need to be tightly regulated to maintain genome stability in human cells.^9^ The need for such a tight regulation of PUMILIO activity might be also the case for the *NORAD*-PUMILIO axis in DDR and could explain why the *NORAD*/PUM1/PUM2 triple KD does not rescue the phenotypes observed in *NORAD* KD cells.

Breast cancer is the most frequently diagnosed malignancy in women, accounting for about one-third of female cancers. Systemic therapies have been shown to be successful in treating early breast cancer; however, once the disease recurs, it tends to be more aggressive and resistant to therapy. The combination of conventional chemotherapeutic agents with novel molecular-targeted agents is a promising therapeutic approach. First, since the targets and mechanisms of action of these agents are different, there is no cross-resistance. Second, in combinatorial approaches, lower concentrations of chemotherapeutic agents may be considered, reducing both their side effects and off-target effects. Third, alterations in expression and/or activity of genes that regulate mitogenic signals caused by molecular-targeted agents may not only disturb cell growth, but also sensitize cancer cells to chemotherapeutic agents.^55^^,^^56^^,^^57^ For example, lncRNA *HOTAIR* contributes to colorectal cancer and 5-FU resistance through the recruitment of EZH2 and subsequent silencing of *miR-218*, upregulation of VOPP1 expression and subsequent activation of the NF-κB/TS pathway.^58^ Similarly, *H19* lncRNA plays a leading role in breast cancer chemoresistance, mediated mainly through a *H19*-*CUL4A*-*ABCB1*/*MDR1* pathway. *H19* expression was greatly upregulated in doxorubicin-resistant breast cancer cells (MCF-7) and its KD sensitizes them to chemotherapy.^59^

Our results show that the DNA damage induced by doxorubicin is enduringly signaled by γH2AX in the absence of *NORAD*. Faulty DNA damage signaling may be caused by an error downstream or upstream of *NORAD*. Upon DNA damage, SAM68 is recruited and stimulates the catalytic activity of PARP1.^60^ Defective ATM activity and reduced γH2AX foci formation in response to γ-irradiation were observed in PARP1-deficient cells. In a *NORAD* KD background as presented here, PARP1 inhibition leads to an increase in γH2Ax deposition, demonstrating a synergistic role of *NORAD* and PARP1 in DDR. In addition, PARP1 is thought to recruit Nbs1 and Mre11 to DSBs in a γH2AX- and MDC1-independent manner.^61^ It is important to know exactly at which point the DNA damage signaling is compromised, especially considering that PARP inhibitors are currently used in patients with advanced-stage breast cancer, in the context of germline mutations in *BRCA1* or *BRCA2* genes, which frequently belong to the triple-negative subtype.^62^

In summary, we demonstrated that *NORAD* confers resistance of breast cancer cells to a chemotherapeutic agent. Therefore, *NORAD* may represent an actionable molecular target and could be used in a combinatorial approach.

## Materials and methods

### Cell lines and culture conditions

The following human breast cell lines and control were used in this study: MCF-10A (non-tumoral, mammary epithelial cell line), MCF-7 (breast carcinoma cell line, luminal A subtype), and MDA-MB-231, MDA-MB-436, and MDA-MB-468 (breast carcinoma cell lines, triple-negative subtype). The MCF-10A cell line was cultured in DMEM/F-12 (Dulbecco’s modified Eagle’s medium/nutrient mixture F-12, Gibco by Life Technologies), supplemented with 5% (v/v) horse serum, epidermal growth factor (20 ng/mL), hydrocortisone (0.5 μg/mL), cholera toxin (100 ng/mL), insulin (10 μg/mL), and 1% (v/v) penicillin-streptomycin. The MCF-7, MDA-MB-231, MDA-MB-436, and MDA-MB-468 cell lines were cultured in DMEM (Gibco by Life Technologies), supplemented with 10% (v/v) heat-inactivated fetal bovine serum and 1% (v/v) penicillin-streptomycin. All cell lines were grown under adherent conditions at 37°C in a humidified incubator with 5% CO_2_. MCF-10A and MCF-7 cell lines were a kind gift from Dr. Sérgio de Almeida (Instituto de Medicina Molecular João Lobo Antunes, Lisbon, Portugal), while MDA-MB-231, MDA-MB-436, and MDA-MB-468 were generously offered by Dr. Sérgio Dias (Instituto de Medicina Molecular João Lobo Antunes, Lisbon, Portugal).

### Obtaining patient tissue samples

After study approval (Project “Ref^a^CE – JMS/is – Estudo 64”) by the Ethics Committee of CUF Descobertas Hospital (Lisbon, Portugal), a retrospective analysis of breast cancer cases diagnosed between 2019 and 2021 at the Pathology Department (CUF Descobertas Hospital) was performed. Clinicopathological information was retrieved, and both hematoxylin and eosin-stained and immunohistochemistry (IHC) slides from selected cases were reviewed by a pathologist with experience in breast pathology. After validating tissue quality and confirming diagnosis, representative tumor and control sections were obtained from FFPE samples.

### RNAscope in tissue samples

Selected FFPE samples were cut to 3 μm sections on positively charged slides. For the RNAscope of the clinical tissue samples, the manufacturer protocol for RNAscope 2.5 Assay was followed, starting with FFPE drying in an oven at 60°C for 1 h. Sequential incubations in xylene and 100% alcohol, accompanied by air drying, were used to deparaffinize the sections. Next, RNAscope Hydrogen Peroxide was applied for 10 min at room temperature (RT), and the slides were rinsed. The RNAscope 1X Target Retrieval Reagent was used for target retrieval for 15 min at 100°C, following standard instructions. After rinsing, the slides were incubated in 100% alcohol for 3 min, and dried at RT, and the tissue area delimited using an Immedge hydrophobic barrier pen to delimit the tissue section. In the HybEz Humidity Control Tray, the slides were incubated with RNAscope Protease Plus at 40°C for the standard time of 30 min. At this point, the tissues were incubated with the probes that could be hybridized to the negative (dapB) or positive (PPIB) controls, or *NORAD* itself. This took place in a HybEz Oven for 2 h at 40°C. The kit contained probes for six sequential amplifications to amplify the hybridization signal. The signal was then ready to be detected after incubating with the Fast RED solutions mix for 10 min at RT. Fast Green Stain Solution (Thermo Scientific, 88024) was applied to the slides. To mount the samples, 1–2 drops of VectaMount Permanent Mounting Medium (Vector Laboratories, H-5000-60) were placed on the slides and the coverslips were dipped in xylene and placed over the sections carefully. Once dry, the samples were evaluated in Nikon ecliplse Ti-U, an inverted wide-field microscope with a CCD color digital camera, at 10× magnification (Achno ADL objective).

### IHC in tissue samples

Tissue sections with a thickness of 3 μm were cut from FFPE samples to positively charged slides for IHC with p53 (clone DO-7, Roche, Switzerland, cat. no. 800–2912) and Ki67 (clone 30-9, Roche, cat. no. 790–4286) antibodies. All IHC was performed on the Ventana BenchMark ULTRA automated staining platform. Antibodies were pre-diluted and run using the OptiView DAB IHC Detection Kit (Roche, cat. no. 760-700) with ULTRA CC1 antigen retrieval (Roche, cat. no. 950-224), and slides evaluated using the optical microscope Leica DM1000 Led, at 200× magnification.

### LNA GapmeR and siRNA transfection

*NORAD* downregulation was performed using RNase H-activating LNA GapmeRs (Exiqon), consisting of chimeric antisense oligonucleotides that contain a central block of DNA, which activates RNase H-dependent cleavage of complementary RNA targets, and are flanked by modified nucleotides (hence LNA [locked nucleic acid]) to offer higher protection of the oligonucleotides against nuclease degradation^63^^,^^64^ and siRNA (Table 1).

**Table 1:** List of siRNAs and Gapmers

siRNAs			
Target mRNA	Sequence (5′–3′)	Reference	
*NORAD*	CUGUGUAUAUAGCGGACAA	siRNA N038095-17	Lincode SMARTpool Human LOC647979 038095-00-0010 (Dharmacon)
	CAUCUAAGCUUUACGAAUG	siRNA N038095-18	
	AGUGCACAAUGUAGGUUAA	siRNA N038095-19	
	CGACCCAAGCCUCGACGAA	siRNA N038095-20	
PUM1	GGUCAGAGUUUCCAUGUGA	siRNA J-014179-05	ON-TARGETplus SMARTpool L-014179-00-0005 (Dharmacon)
	GGAGGAGGCGGCUAUAAUA	siRNA J-014179-06	
	GGAGAUAAGCUAGGAGAUU	siRNA J-014179-07	
	CGGAAGAUCGUCAUGCAUA	siRNA J-014179-08	
PUM2	CUGAAGUAGUUGAGCGCUU	siRNA J-014031-17	ON-TARGETplus SMARTpool L-014031-02-0005 (Dharmacon)
	GCAGAGUAAUUCAGCGCAU	siRNA J-014031-18	
	GACAAAUGGUAGUGGUCGA	siRNA J-014031-19	
	AGACAUAACAGUAACACGA	siRNA J-014031-20	

Cells were transfected with either control (non-specific) LNA GapmeRs or LNA GapmeRs directed against *NORAD*, using Lipofectamine RNAiMAX transfection reagent (Invitrogen), with 24 or 48 h between the two transfections, following standard procedures (final concentration of 25 nM). Two different GapmeRs were designed using the Antisense LNA GapmeR design tool, with the following central sequences: 5′-CTAGACGTAAATTAGG-3’ (human *NORAD* GapmeR 1) and 5′- ACTTTACTAAAAACGC-3’ (human *NORAD* GapmeR 2). siRNAs used for NORAD, PUM1 and PUM2 were the same as in Tichon et al.^12^ KD efficiency was assessed by qRT-PCR. siRNAs used for PARP1 (Santa Cruz, sc-29437) and CDK1 (Thermo Fisher Scientific, AM16704 103821) were used at 25 nM. KD efficiency was assessed by qRT-PCR and WB. A combination of unspecific siRNA + scrambled LNA Gapmer was used as a control at the same concentrations.

### Single-molecule RNA FISH

Stellaris FISH probes recognizing *NORAD* and labeled with Quasar 570 dye were purchased from Biosearch Technologies. The probe set sequences utilized in the experiments had been described previously and each set comprises 48 different oligonucleotides (20 nucleotides in length).^9^

Cells were seeded on gelatin-coated glass coverslips in flat-bottom 24-well cell culture plates (TPP), washed in phosphate-buffered saline (PBS), and fixed with 3.7% paraformaldehyde for 10 min at RT. Fixed cells were then washed in PBS, permeabilized in 70% ethanol for 1 h at RT, and washed with a solution containing 20× saline sodium citrate (SSC), deionized formamide, and nuclease-free water. Within a humidified chamber, coverslips were transferred onto drops of hybridization buffer (containing probe, 50% dextran sulfate, 20× SSC, deionized formamide, 100% formaldehyde, and nuclease-free water), and hybridized overnight at 37°C. Coverslips were washed with the previsouly detailed buffer and with 2× SSC, following which they were mounted with VECTASHIELD and 4′,6-diamidino-2-phenylindole mounting medium. Images were acquired using a laser scanning confocal inverted microscope (LSM710, Carl Zeiss).

### Cellular viability assay

Cells were seeded in 48-well plates (TPP), at a density of 20,000–60,000 cells/well and incubated at 37°C in a humidified incubator with 5% CO_2_. When applicable, *NORAD* downregulation was performed at the time of seeding and 24 h later, as mentioned above or in the figure legend.

Seventy-two hours after plating, cells were incubated with doxorubicin (Sigma-Aldrich, D2975000) for 24 h, in a range of concentrations. Specific culture medium containing 10% (v/v) alamarBlue Cell Viability Reagent (Thermo Fisher Scientific) was then added to the cells and resazurin assay performed. Plates were incubated for 2 h at 37°C protected from light and the fluorescence intensity was then quantified using a plate-reading fluorometer (Microplate Reader Infinite M200, Tecan) with excitation wavelength at 560 nm and emission wavelength at 590 nm. The relative viable cell number was standardized to untreated cells and the IC_50_ for each drug determined from dose-response curves using GraphPad Prism software.

### Cell apoptosis analysis

Analysis of apoptosis was performed using the Annexin V Apoptosis Detection Conjugate (Thermo Fisher Scientific, A35110). Cells were trypsinized, centrifuged at 1,200 rpm for 5 min, washed with 1× PBS and resuspended in 1× Binding Buffer Solution at a final concentration of 1 × 10^6^ cells/mL. To each 100 μL of cell suspension were added 2.5 μL of Annexin V-CF blue conjugate and 5 μL of 7-AAD staining solution. After incubation at RT for 15 min in the dark, 400 μL of 1× binding buffer solution was added, cells were transferred to FACS tubes and analyzed in a BD LSRFortessaTM X-20 cytometer. Results were analyzed using the FlowJo software.

### qPCR analysis of gene expression

Total RNA was isolated using NZYol following manufacturer’s instructions (NZYTech). RNA quality was verified using a NanoDrop 2000 Spectrophotometer (Thermo Fisher Scientific). cDNA was synthesized with random primers using the Roche Transcriptor High Fidelity cDNA Synthesis Kit. qRT-PCR analysis was performed in the ViiA 7 Real-Time PCR System (Thermo Fisher Scientific) using SYBR Green PCR master mix (Thermo Fisher Scientific). Gene-specific primer pairs (Sigma) were used as follows:

*NORAD* forward 5′-TGTTTGTGCAGTGGTTCAGG-3′

reverse: 5′-TCTTGCCTCGCTGTAAACAG-3′

p53 forward: 5′-CCCCTCCTGGCCCCTGTCATCTTC-3′

reverse: 5′-GCAGCGCCTCACAACCTCCGTCAT-3′

18s forward: 5′-GGATGTAAAGGATGGAAAATACA-3′

reverse: 5′-TCCAGGTCTTCACGGAGCTTGTT-3′

GAPDH forward: 5′-GACAGTCAGCCGCATCTTCT-3′

reverse: 5′-TTAAAAGCAGCCCTGGTGAC-3′

PUM1 forward: 5′- CCGGGCGATTCCTGTCTAA-3′

reverse: 5′- CCTTTGTCGTTTTCATCACTGTCT-3′

PUM2 forward: 5′- GGGAGCTTCTCACCATTCA-3′

reverse: 5′- CCATGAAAACCCTGTCCAGATC-3′

MCM6 forward: 5′- GAGGAACTGATTCGTCCTGAGA

reverse: 5′- CAAGGCCCGACACAGGTAAG

PARP1 forward: 5′-GCAGAGTATGCCAAGTCCAACAG-3′

reverse: 5′-ATCCACCTCATCGCCTTTTC-3′

BUB3. forward: 5′- GGTTCTAACGAGTTCAAGCTGA

reverse: 5′- GGCACATCGTAGAGACGCAC

Relative fold changes in gene expression were calculated based on the threshold cycle (Ct), using the 2^−ΔΔCt^ method, considering GAPDH exclusively or in combination with 18S ribosomal RNA as endogenous controls.

### Correlation analysis between *NORAD* expression and survival: KM Plotter Online

The open access KM Plotter Online Tool was used to explore the association between *NORAD* expression and the clinical outcome for breast cancer patients, namely overall survival and relapse-free survival.^32^^,^^65^ This platform integrates information available at Gene Expression Omnibus, European Genome-phenome Archive and The Cancer Genome Atlas, incorporating high-throughput data with clinical information.^32^^,^^65^ After selecting the genes of interest and the characteristics of the study sample, a Kaplan-Meier survival curve, the hazard ratio with 95% confidence intervals, and log rank p values are displayed for each combination.

### Sample preparation for spectrometric analysis

Samples (10 μg) were reduced with dithiothreitol (30 nmol, 37°C, 60 min) and alkylated in the dark with iodoacetamide (60 nmol, 25°C, 30 min). The resulting protein extract was diluted to 2 M urea with 200 mM ammonium bicarbonate for digestion with endoproteinase LysC (1:10 w:w, 37°C, 6 h, Wako, cat. no. 129–02541), and then diluted 2-fold with 200 mM ammonium bicarbonate for trypsin digestion (1:10 w:w, 37°C, o/n, Promega cat. mo. V5113).

After digestion, peptide mix was acidified with formic acid and desalted using a MicroSpin C18 column (The Nest Group) prior to LC-MS/MS analysis.

### LC-MS

Samples were analyzed using an LTQ-Orbitrap Fusion Lumos mass spectrometer (Thermo Fisher Scientific, San Jose, CA) coupled to an EASY-nLC 1200 (Thermo Fisher Scientific [Proxeon], Odense, Denmark). Peptides were loaded directly onto the analytical column and were separated by reversed-phase chromatography using a 50 cm column with an inner diameter of 75 μm, packed with 2 μm C18 particles (Thermo Scientific).

Chromatographic gradients started at 95% buffer A and 5% buffer B with a flow rate of 300 nL/min for 5 min and gradually increased to 25% buffer B and 75% A in 79 min and then to 40% buffer B and 60% A in 11 min. After each analysis, the column was washed for 10 min with 10% buffer A and 90% buffer B. Buffer A: 0.1% formic acid in water. Buffer B: 0.1% formic acid in 80% acetonitrile.

The mass spectrometer was operated in positive ionization mode with nanospray voltage set at 2.4 kV and source temperature at 305°C. The acquisition was performed in data-dependent acquisition mode and full MS scans with one micro scan at resolutions of 120,000 were used over a mass range of *m*/*z* 350–1,400 with detection in the Orbitrap mass analyzer. Auto gain control (AGC) was set to “standard” and injection time to “auto.” In each cycle of data-dependent acquisition analysis, following each survey scan, the most intense ions above a threshold ion count of 10,000 were selected for fragmentation. The number of selected precursor ions for fragmentation was determined by the “Top Speed” acquisition algorithm and a dynamic exclusion of 60 s. Fragment ion spectra were produced via high-energy collision dissociation at a normalized collision energy of 28% and acquired in the ion trap mass analyzer. AGC was set to 2E4, and an isolation window of 0.7 *m*/*z* and a maximum injection time of 12 ms were used.

Digested bovine serum albumin (New England Biolabs, cat. no. P8108S) was analyzed between each sample to avoid sample carryover and to assure stability of the instrument, and QCloud^66^ was used to control instrument longitudinal performance during the project.

### Proteomic data analysis

The LC-MS/MS raw files were elaborated using MaxQuant (v.1.6.17.0) for the processes of protein identification and quantification according to the LFQ algorithm.^67^^,^^68^ Runs were analyzed using the Andromeda search engine against the freely available reference proteome of *Homo* *sapiens* downloaded from the UniProtKB database (January 2021). The allowable tolerance for precursor mass and fragment mass was set at 4.5 and 20 ppm, respectively. The minimum peptide length was set at seven amino acids and trypsin and LysC were selected as the proteolytic enzyme allowing up to two missing cleavage sites. Carbamidomethylation (Cys) was set as the fixed modification, while oxidation (Met), deamidation (ND), and N-terminal protein acetylation were the variable modifications. The false discovery rate was set at 1% at both the protein and peptide levels. In this analysis, the inter-run agreement option was selected. According to the MaxLFQ algorithm, proteins were quantified based on the extracted ion currents of the precursor ion peptides. The results of this analysis were first imported into Perseus (v.1.6.14.0) and then into MetaboAnalyst 5.0 for univariate and multivariate statistical data analysis and visualization. In brief, proteins identified as site only, reverse, and contaminants were removed. Expression values were transformed to a logarithmic scale with base 2. Samples were annotated according to their respective groups. Abundance of proteins between two groups were compared using a two-tailed t test, with the adjusted p value set at <0.05. Principal-component analysis (PCA) was performed on the matrix before logarithmic transformation; after filtering valid values, a multistream plot and histogram were generated; after the two-sample t test, a volcano plot was generated. PLSDA and variable importance in projection from the previous analysis were extracted. A heatmap was performed from the top 100 proteins, the 2 clusters (downregulated and upregulated) were extracted and filtered according to the PCA values (>2). Resulting clusters were run on STRING and g:PROFILER to explore the biological functions of the proteins.

The MS proteomics data have been deposited to the ProteomeXchange Consortium via the PRIDE^69^ partner repository with the dataset identifier PXD039920.

### WB analysis

Proteins were extracted on ice after cell washing in PBS (Fisher Bioreagents, BP399-1), with RIPA buffer containing protease and phosphatase inhibitors (RIPA, Thermo Scientific, 89901; EDTA 100×, Thermo Scientific, 1861275; Cocktail protease inhibitor 100×, Thermo Scientific, 1861278; Cocktail phosphatase inhibitor 100×, Thermo Scientific, 1861277). Cell lysates were incubated on ice for 30 min and centrifuged at 15,000 × *g* at 4°C for 15 min. Protein levels were evaluated using the BCA assay according to the manufacturer’s protocol (Pierce BCA Protein Assay Kit, Thermo Scientific, 23227). Thirty micrograms of protein from each sample was prepared for loading in NuPAGE LDS Sample Buffer (Invitrogen, NP0004) and separated in a precast gel (Bolt 4%–12%, Bis-Tris, 1.0 mm, Mini Protean Gels, NW04120Box, Thermo Scientific; Running Buffer: 20× Bolt MES SDS Running Buffer, B0002, Thermo Scientific). After wet transfer, the nitrocellulose membranes were stained with Ponceau S (0.1%, w/v) for 15 min to assess gel loading. Prior to immunoblotting, membranes were blocked with 5% bovine serum albumin (BSA), prepared in TBS-T, and then incubated with the indicated antibodies. Membranes were visualized in a chemiluminescence-based system (ChemiDoc Touch [Bio-Rad]), and protein levels were calculated using the Ponceau S staining for normalization. For a list of the antibodies used see Table 2.

**Table 2:** – List of antibodies

Antibody	Type	Reference	Dilution
γH2AX	primary antibody	ab2893 (Abcam)	1:1,000
PUM2	primary antibody	ab92390 (Abcam)	1:10,000
H2AX	primary antibody	10856-1-AP (Proteintech)	1:2,000
PARP1	primary antibody	MA3-950 (Thermo Fisher Scientific)	1:500
CDK1	primary antibody	Ab131450 (Abcam)	1:500
Anti-mouse	cross-adsorbed secondary antibody, HRP	G21040 (Invitrogen)	1:10,000
Anti-rabbit	cross-adsorbed secondary antibody, HRP	G21234 (Invitrogen)	1:10,000

### Immunofluorescence

Cells previously seeded on 24-well plates in coverslips with gelatin coating were prepared according to the different experimental conditions (see figure legends) and fixed with 4% formaldehyde for 20–25 min, washed with PBS, and permeabilized (0.2% Triton X-100 in PBS) for 10 min. Samples were then washed three times with PBS and blocked for 1 h in PBS containing 1% BSA, incubated overnight at 4°C with primary antibodies diluted in PBS containing 1% BSA, washed with PBS containing 0.05% Triton X, and incubated for 2 h at RT with secondary antibodies conjugated with Alexa 647 (diluted in PBS with 1% BSA), and finally washed again with 0.01% Triton X. Images were acquired using a Zeiss confocal microscope (Zeiss LSM 880).

### Wound healing assay

MDA-MB-231 and MDA-MB-468 cells were seeded into 24-well plates and grown to sub-confluence. Cell proliferation was blocked by a 2 h pre-treatment with mitomycin C (100 ng/mL) in serum-free medium. A scratch was made in each well using a 1,000 μL pipette tip and the wounded monolayers washed twice with PBS to remove cell debris and floating cells. Wound width was monitored over time (see corresponding images and figure legends) under an inverted microscope with a digital camera. Percentage wound recovery was expressed compared with the width of the wound at t = 0 (100%).

### Comet assay

MDA-MB-231 cells were seeded and exposed to the different experimental conditions as depicted in the corresponding pictures. For the Comet assay we follow the manufacturer’s protocol (Fischer Scientific, 13464434). Tail DNA analysis was processed with the Cometscore 2.0 software.

### Statistical analysis

Results are presented by the mean along with the standard deviation, and respective p value. Kruskal-Wallis (medians of three or more independent groups) and Mann-Whitney (comparison of two groups) tests were used to calculate statistical significance. A log rank test was used to calculate the statistical differences in the survival curves (KM Plotter Online Tool). Statistical power is detailed in the corresponding figure legends.

## Data Availability

Data available on request from the authors.

## Supplemental information

Document S1. Figures S1–S9

Document S2. Article plus supplemental information
