# Supplementary material for: Expression of NORAD correlates with breast cancer aggressiveness and protects breast cancer cells from chemotherapy
Source: Mol Ther Nucleic Acids. 2023 Aug 18;33:910–24. doi: 10.1016/j.omtn.2023.08.019 (PMC10480464; doi:10.1016/j.omtn.2023.08.019)
Supplement: Document S1. Figures S1–S9 [file mmc1.pdf]

OMTN, Volume *33*

Supplemental information

Expression of *NORAD* correlates with breast cancer aggressiveness and protects breast cancer cells from chemotherapy

Catarina Alves-Vale, Ana Maria Capela, Carlota Tavares-Marcos, Beatriz Domingues-Silva, Bruno Pereira, Francisco Santos, Carla Pereira Gomes, Guadalupe Espadas, Rui Vitorino, Eduard Sabidó, Paula Borralho, Sandrina Nóbrega-Pereira, and Bruno Bernardes de Jesus

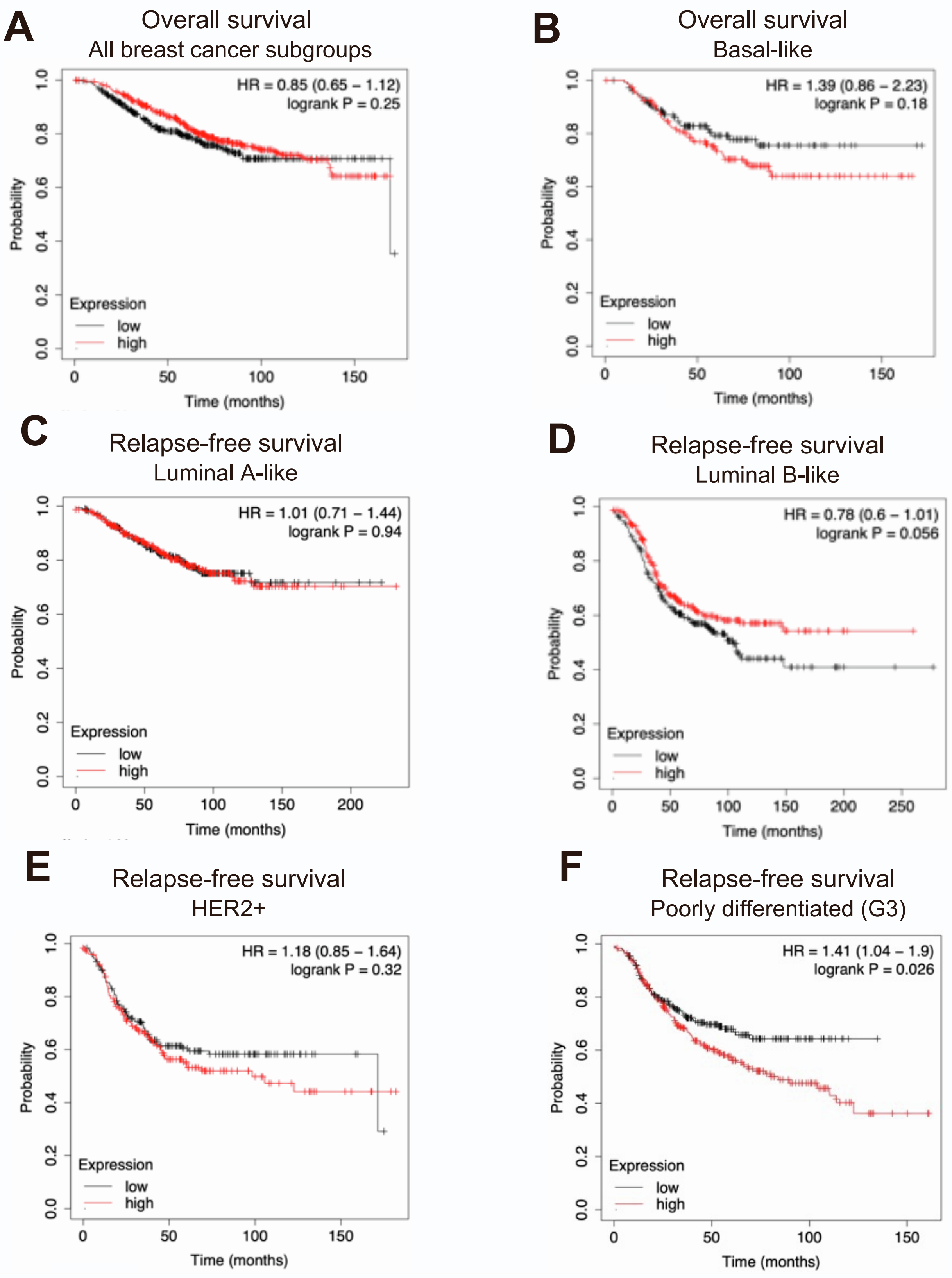

Figure S1 – Survival curves for breast cancer patients grouped by their NORAD levels (Kaplan-Meier Plotter): (a) Overall survival for all breast cancer subtypes, (b) Overall survival for basal-like breast cancer, (c-e) Relapse-free survival for specific subtypes of breast cancer: Luminal A-like (c), Luminal B-like (d), and HER2+ (e), (f) Relapse-free survival for poorly differentiated (grade 3) breast cancer. The curves show the probability of survival over time and are coloured based on the NORAD levels. The x-axis represents time in months, and the y-axis represents the proportion of patients who are still alive and free from relapse.

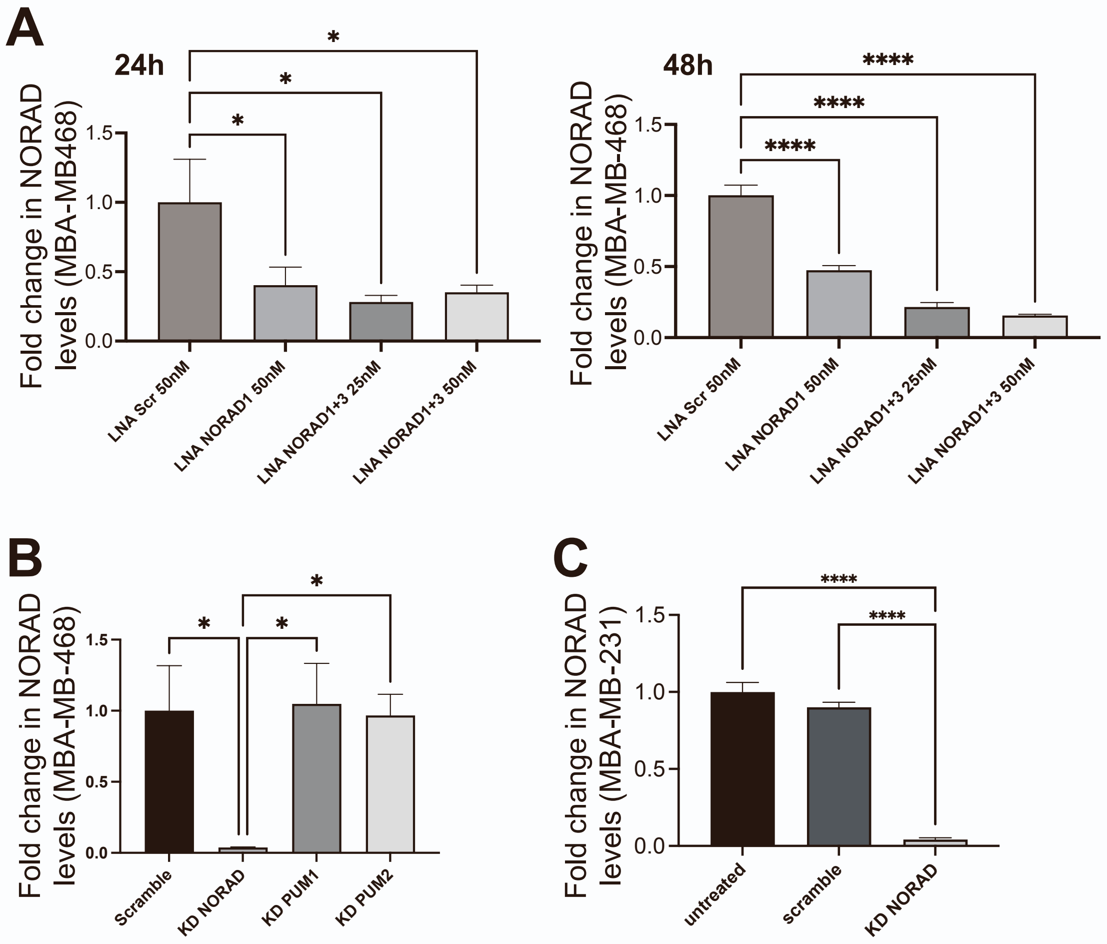

Figure S2 – NORAD levels after LNA or combinatorial LNA+siRNA approaches: (a) *NORAD* mRNA levels after knockdown, using LNA^{TM} GapmeRs, with 24h or 48h interval between transfections, in the MDA-MB-468 cell line (RT-qPCR: GAPDH as housekeeping gene, n=3); (b) *NORAD,* PUM1 and PUM2 mRNA levels after knockdown, using LNA^{TM} GapmeRs and siRNAs, with 24h interval between transfections, in the MDA-MB-468 cell line (RT-qPCR, GAPDH as housekeeping gene, n=3). (c) *NORAD* mRNA levels after knockdown, using LNA^{TM} GapmeRs and siRNAs, with 24h interval between transfections, in the MDA-MB-231 cell line (RT-qPCR, GAPDH as housekeeping gene, n=3). No-symbol p>0.05, * p<0.05, ** p<0.01 and ***p<0.001.

Figure S3

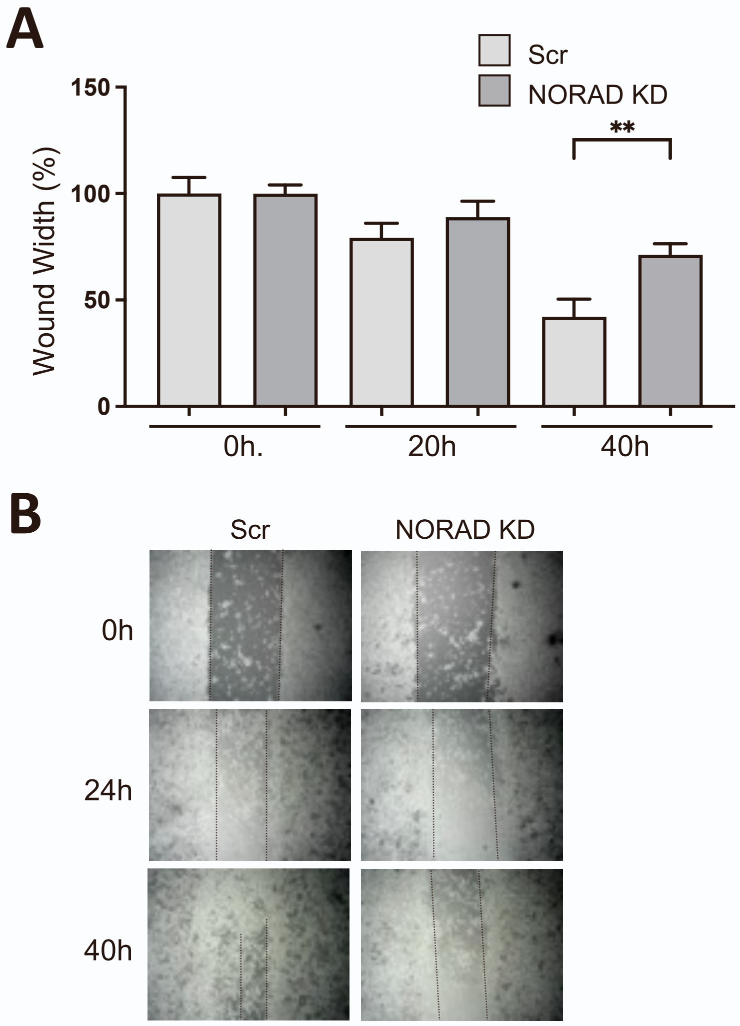

Figure S3 – (a,b) NORAD knockdown effect on cell migration, in the MDA-MB-468 cell line (wound healing assay), (a) is the gap quantification at the indicated timepoints (n=3), (b) is a representative image of the wound healing. No-symbol p>0.05, * p<0.05, ** p<0.01 and ***p<0.001.

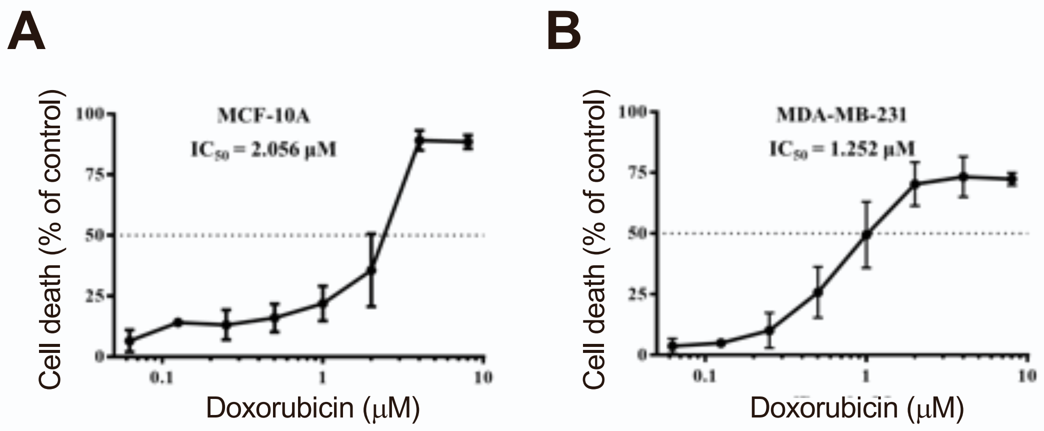

Figure S4 – IC50 determination in the MCF-10a and MBA-MD-231 cell lines. AlamarBlue® reduction assay was performed and doxorubicin IC50 was determined for the mentioned cell lines.

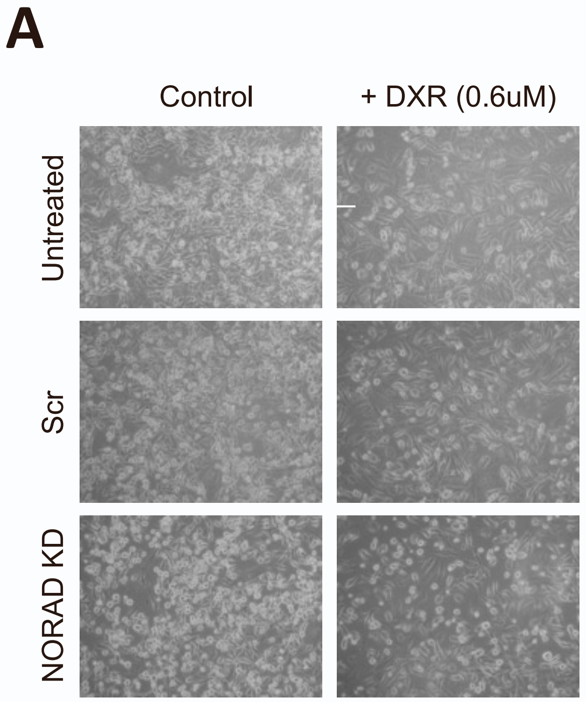

Figure S5 – Representative image of MDA-MB-231 cells in the depicted conditions.

A

Biological Process (Gene Ontology)			
GO-term	Description	Count in network	False discovery rate
GO:0000727	Double-stranded break repair via break-induced replication	3 of 11	0.00073
GO:0000082	G1/S transition of mitotic cell cycle	9 of 128	2.37e-08
GO:0031571	Mitotic g1 DNA damage checkpoint	4 of 67	0.0023
GO:0000724	Double-strand break repair via homologous recombination	4 of 102	0.0084
GO:0071156	Regulation of cell cycle arrest	4 of 110	0.0099
GO:2000045	Regulation of g1/s transition of mitotic cell cycle	5 of 156	0.0026
GO:0010389	Regulation of g2/m transition of mitotic cell cycle	5 of 198	0.0068
GO:0006281	DNA repair	10 of 522	1.42e-05
GO:1901990	Regulation of mitotic cell cycle phase transition	7 of 416	0.0023
GO:0006974	Cellular response to DNA damage stimulus	12 of 793	5.95e-06
GO:0033554	Cellular response to stress	13 of 1725	0.0014

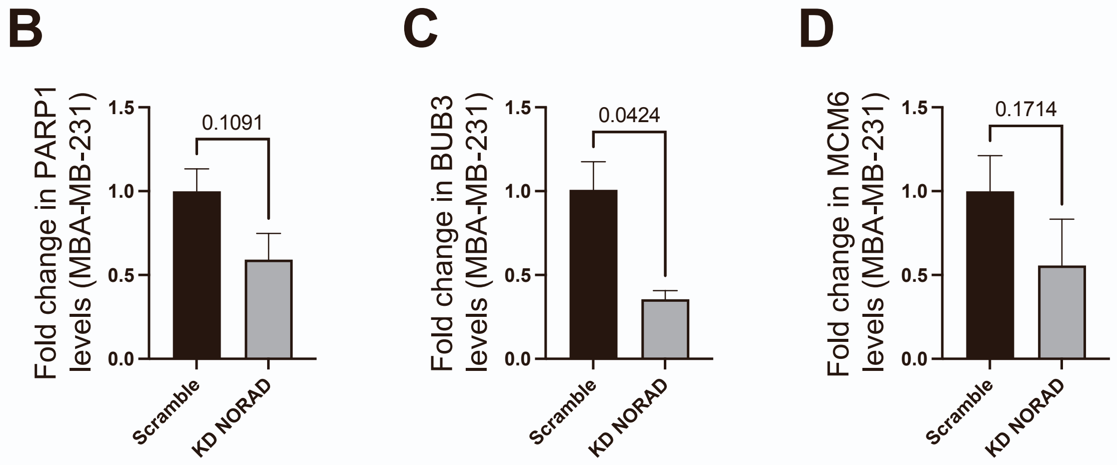

Figure S6 – (a) Gene ontology analysis (biological processes) of the top genes downregulated in the NORAD-KD MDA-MB-231 cell line. (b-d) PARP1, BUB3 and MCM6 mRNA levels in the MDA-MB-231 cell line treated with the depicted conditions (n=3).

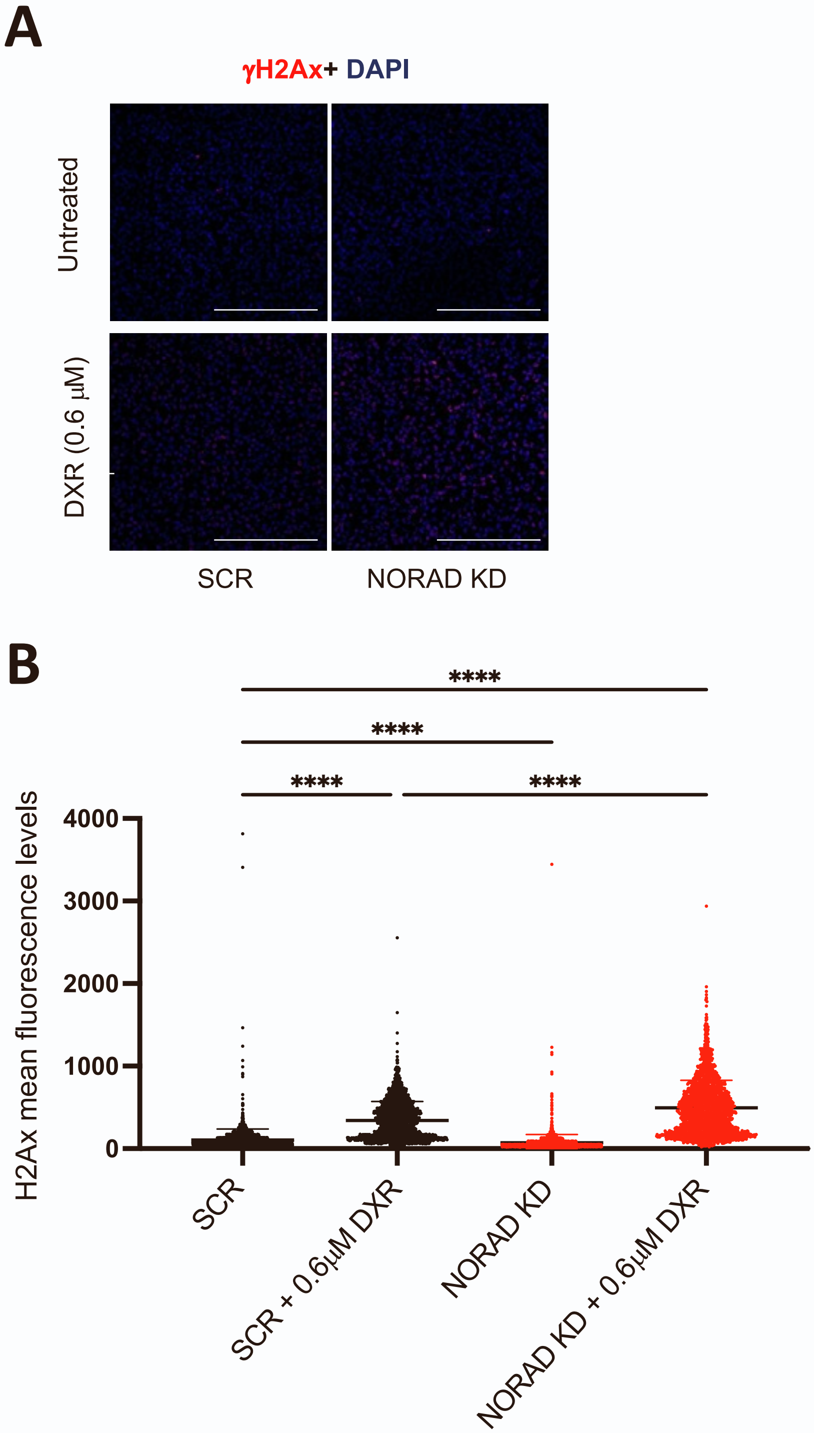

Figure S7 – (a) Immunofluorescence for γH2Ax in the depicted experimental conditions, in the MDA-MB-231 cell line; (b) Quantification of the signal corresponding to γH2Ax in the depicted experimental conditions, in the MDA-MB-231 cell line (scale bar corresponds to 300um, for details see material and methods). No-symbol p>0.05, * p<0.05, ** p<0.01 and ***p<0.001.

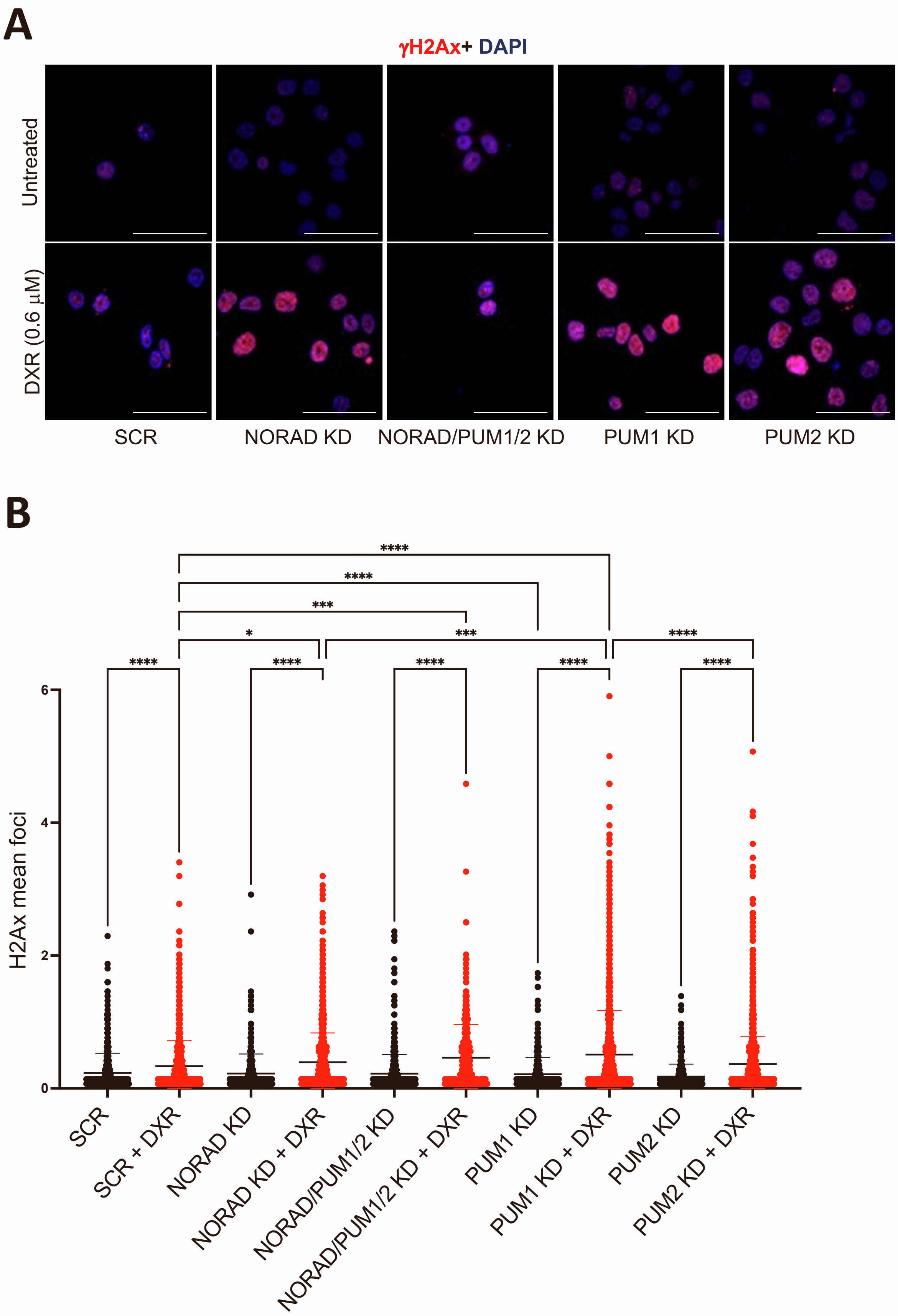

Figure S8 – (a) Immunofluorescence for γH2Ax in the depicted experimental conditions, in the MDA-MB-468 cell line; (b) Quantification of the signal corresponding to γH2Ax in the depicted experimental conditions, in the MDA-MB-468 cell line (scale bar corresponds to 60um, for details see material and methods). No-symbol p>0.05, * p<0.05, ** p<0.01 and ***p<0.001.

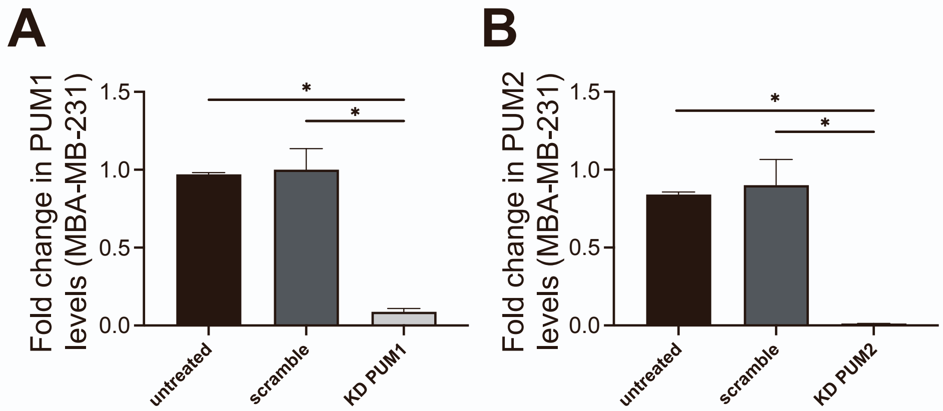

Figure S9 – (a,b) PUMILIO1 and PUMILIO2 mRNA levels in the MDA-MB-231 cell line treated with the depicted conditions (n=3). No-symbol p>0.05, * p<0.05, ** p<0.01 and ***p<0.001.
